# Diagnosing the Periphery: Using the Rey–Osterrieth Complex Figure Drawing Test to Characterize Peripheral Visual Function

**DOI:** 10.1177/2041669517705447

**Published:** 2017-05-29

**Authors:** Daniel R. Coates, Johan Wagemans, Bilge Sayim

**Affiliations:** Laboratory of Experimental Psychology, KU Leuven, Leuven, Belgium; Institute of Psychology, University of Bern, Bern, Switzerland; Laboratory of Experimental Psychology, KU Leuven, Leuven, Belgium; Laboratory of Experimental Psychology, KU Leuven, Leuven, Belgium; Institute of Psychology, University of Bern, Bern, Switzerland; SCALab – Sciences Cognitives et Sciences Affectives, Universités de Lille, Lille, France

**Keywords:** peripheral vision, drawing, crowding, phenomenology

## Abstract

Peripheral vision is strongly limited by crowding, the deleterious influence of neighboring stimuli on target perception. Many quantitative aspects of this phenomenon have been characterized, but the specific nature of the perceptual degradation remains elusive. We utilized a drawing technique to probe the phenomenology of peripheral vision, using the Rey–Osterrieth Complex Figure, a standard neuropsychological clinical instrument. The figure was presented at 12° or 6° in the right visual field, with eye tracking to ensure that the figure was only presented when observers maintained stable fixation. Participants were asked to draw the figure with free viewing, capturing its peripheral appearance. A foveal condition was used to measure copying performance in direct view. To assess the drawings, two raters used standard scoring systems that evaluated feature positions, spatial distortions, and omission errors. Feature scores tended to decrease with increasing eccentricity, both within and between conditions, reflecting reduced resolution and increased crowding in peripheral vision. Based on evaluation of the drawings, we also identified new error classes unique to peripheral presentation, including number errors for adjacent similar features and distinctive spatial distortions. The multifaceted nature of the Rey–Osterrieth Complex Figure—containing configural elements, detached compound features, and texture-like components—coupled with the flexibility of the free-response drawing paradigm and the availability of standardized scoring systems, provides a promising method to probe peripheral perception and crowding.

## Introduction

It is well established that visual resolution in the periphery is inferior to resolution in the fovea, the center of gaze, but a complete understanding of the differences between peripheral and foveal vision is still lacking (Lettvin, 1976; Strasburger, Rentschler, & Jüttner, 2011). The ability to discriminate fine spatial details, such as the gap in a capital letter “C,” worsens in the periphery (Weymouth, 1958), which has often been characterized as “blurriness” (though see Discussion section). Furthermore, tasks such as identifying the spatial location of objects (Michel & Geisler, 2011) as well as tasks involving relative spatial localization (Levi, Klein, & Yap, 1987; Westheimer, 1982) are measurably worse in the visual periphery. Finally, in the last several decades, interest has coalesced around the phenomenon of “crowding,” which is the difficulty identifying objects when they are surrounded by other objects, an effect most pronounced for stimuli located in the periphery (for reviews, see Herzog, Sayim, Chicherov, & Manassi, 2015; Levi, 2008; Pelli et al., 2007; Strasburger et al., 2011; Whitney & Levi, 2011). Many studies have measured quantitative aspects of the performance deterioration due to crowding, such as the size and shape of the region within which flankers impair performance, called the crowding zone (Bouma, 1970; Toet & Levi, 1992). It has been shown that this zone is elliptical, extending radially toward the fovea, and grows approximately linearly with increasing eccentricity (Toet & Levi, 1992). Some studies have examined what basic stimulus factors modulate the magnitude of crowding or the size of the crowding zone, such as display duration (Chung & Mansfield, 2009; Tripathy & Cavanagh, 2002), the spatial complexity of the stimulus (Bernard & Chung 2011), target size (Pelli, Palomares, & Majaj, 2004; Strasburger, Harvey, & Rentschler, 1991; Tripathy & Cavanagh, 2002), or stimulus contrast (Chung, Levi, & Legge, 2001; Coates, Chin, & Chung, 2013; Pelli et al., 2004; Strasburger et al., 1991). Other studies have looked at relationships between target and flanker characteristics, such as the effects of target-flanker similarity (Bernard & Chung 2011; Chung & Mansfield, 2009; Kooi, Toet, Tripathy, & Levi, 1994; Sayim, Westheimer, & Herzog, 2008). Finally, the overall configuration of the target and flankers, including how much the target and the flankers group with each other, plays a key role in crowding (Banks & White, 1984; Livne & Sagi, 2007; Malania, Herzog, & Westheimer, 2007; Manassi, Sayim, & Herzog, 2012, 2013; Sayim et al., 2008; Sayim, Westheimer, & Herzog, 2010, 2011; Sayim & Cavanagh, 2013; Saarela, Sayim, Westheimer, & Herzog, 2009; Sayim, Greenwood, & Cavanagh, 2014).

Typically, these aspects of crowding have been studied using forced-choice methods, which have dominated vision science research, especially since the formalization of signal detection theory (Green & Swets, 1966). Specifically, in order to control for response bias factors, experimental subjects are forced to choose from a set of possible responses for a trial, such as “signal absent” or “signal present,” the orientation of a tilted element, or one of the letters of the alphabet. In addition, experiments typically utilize stimuli such as simple shapes, Roman letters, oriented gratings, or Gabor patches (Levi, 2008). With the combination of forced-choice techniques and impoverished stimuli, important aspects of phenomenological experience are lost. For example, subjects attempting to identify a crowded letter may perceive a visual form that does not match any of the set of possibilities. Similarly, gratings may assume a warped or distorted appearance, a quality that has been observed for very high-frequency (aliased) gratings at the fovea (Williams, 1985) or in amblyopic vision (Barrett, Pacey, Bradley, Thibos, & Morrill, 2003; Hess, Campbell, & Greenhalgh, 1978). Crowded stimuli have been described as “jumbles” of their constituent features (Pelli et al., 2004), with subjects reporting that features seem indeterminately located among the multiple proximal stimuli (Korte, 1923).

The important (but often overlooked) treatise by Korte (1923) is an early study of the phenomenological aspects of word perception in the periphery (summarized in Strasburger, 2014 and Strasburger et al., 2011). In his experiments, Korte carefully noted the impressions of subjects reporting the appearance of indirectly presented words. He enumerated a variety of errors that occurred using qualitative descriptions, such as incorrect absorption of features into adjacent letters or the “buzzing around” of small spatial features that could not be stably localized.

Recent work from our group using crowded peripheral letter-like shapes introduced several error categories observed in crowded displays (Sayim & Wagemans, 2013). In that study, we asked subjects to draw simple letter-like stimuli viewed peripherally, in order to examine the effects of crowding on the perception of shapes comprising several line segments. Using these simplified stimuli and a free-response paradigm yielded meaningful parametric stimulus dimensions and permitted the quantification of explicit error types, such as the diminishment of target features.

Observer drawings have been used to illustrate effects such as the grating distortions described earlier (Barrett et al., 2003; Hess et al., 1978; Williams, 1985) or as demonstrations of crowded percepts (Pelli & Tillman, 2008), but few researchers have quantitatively evaluated drawings themselves. Johnson and Uhlarik (1974) asked subjects to draw geometric shapes such as squares and triangles presented briefly in the fovea, and evaluated the presence or absence of edges in the drawings to probe the microgenesis of visual perception. We have analyzed drawings of crowded letters and letter-like shape (Sayim & Wagemans, 2013) and used a drawing method to investigate the effects of prior knowledge on crowded color appearance in the periphery (Sayim, Myin, & Van Uytven, 2015).

In clinical neuropsychology, on the other hand, drawing tasks are heavily used, for a variety of reasons. Subjective experience is of primary interest, as patients may have completely unexpected interpretations of their visual input. Many drawing tests are straightforward, typically requiring the copying of a geometric shape, and easy to administer, requiring only score sheets and pens. Standardization and scoring is the primary challenge that has faced clinical researchers. A variety of drawing tasks have been introduced, including the Rey–Osterrieth Complex Figure (ROCF; Osterrieth, 1944; Rey 1941) and the Taylor Complex Figure (Taylor, 1969). Here, we focus on the ROCF, which is the most widely used drawing figure in neuropsychological practice (for a recent review, see Shin, Park, Park, Seol, & Kwon, 2006). Evaluation of drawings of this figure have been shown to reveal deficits in visual-spatial processing and visual working memory (Shin et al., 2006), including particular types of spatial errors that are sensitive to hemispheric lateralization of lesions (Loring, Lee, & Meador, 1988). In addition, the organizational strategies used to draw these figures can reveal subtle aspects of development maturity in children (Waber & Holmes, 1985, 1986), including a shift in preference from local features to global aspects of the figure.

Many of these same issues in visual-spatial processing (such as spatial mislocalization errors, local vs. global distortions, different types of integration problems, etc.) are also germane to the study of peripheral vision. To understand the nature and limits of peripheral vision requires more than the measurement of performance in identification tasks. In particular, it is important to understand the characteristics, for example, the types of distortions exhibited in the perception of complex spatial forms viewed peripherally. Several results using traditional stimuli and forced-choice methods have revealed clues that target appearance is modulated by flankers in crowded conditions.

For example, one study (Greenwood, Bex, & Dakin, 2010) used noise “targets”—themselves lacking distinctive features—flanked by oriented Gabors, showing assimilation of the flanker orientation and tilt aftereffects from adaptation. These authors concluded that the flankers biased target appearance as a general-purpose mechanism in the periphery that regularizes adjacent spatial forms. Similarly, Sayim and Cavanagh (2013), using rotated letter targets and flankers, showed that flankers perceptually bias target appearance by shape-specific assimilation, in a dissociable fashion for crowding and grouping. How to extrapolate these results to more complex multidimensional targets is unclear, since the structure of such stimuli may resist decomposition based on averaging or assimilation.

Peripheral vision has sometimes been described as statistical summarization in terms of texture (Balas, Nakano, & Rosenholtz, 2009; Freeman & Simoncelli, 2011). This interpretation is likely relevant for many aspects of visual perception, such as natural scenes, but not necessarily for all stimuli, such as alphabetic text or well-defined objects and shapes. We are interested in the perception of both “textures” and “objects,” or equivalently “stuff” and “things” (Adelson & Bergen, 1991).

Therefore, we used the rich, multipart ROCF to study peripheral perception directly by asking subjects to portray what they saw, using drawing. While line drawings are generally suited to depict a large range of visual features (Sayim & Cavanagh 2011), the ROCF has the particular advantage of containing both object-like and texture-like components. It comprises a variety of elements in nontrivial configurations, unlike stimuli that are simple figures like Gabors, gratings, or simple geometric figures. In contrast to studies with simple stimuli, the phenomenon known as “internal crowding” can be revealed. Internal crowding, or “within-object” crowding, refers to the effect whereby the parts of a complex object crowd each other, an effect that has been observed with cartoon faces (Martelli, Majaj, & Pelli, 2005) and Chinese characters (Zhang, Zhang, Xue, Liu, & Yu, 2009). Complicated multipart objects, such as the ROCF, are essential to show this effect, rather than simple letter-like stimuli with few edges. While the deleterious effects of within-object crowding is clear (as shown by the previous studies), more detailed understanding of what “happens” to the parts, such as whether they can be duplicated, or whether the overall form loses all organization, remains lacking.

As far as we know, this is the first time this clinical instrument has been used to systematically study the spatial distortions of form perception in the periphery of normal subjects. Although our intent is not to make a link between peripheral vision and cognitive impairment, our goals are surprisingly similar to clinical researchers. Specifically, we seek to find specific patterns of errors that reveal differences in visual perception between conditions—in our case location in the visual field. However, whereas the primary purpose of clinical instruments is to provide differential diagnoses, our focus is on investigating the spatial distortions themselves, in order to better understand the underlying mechanisms of peripheral vision.

Clinical researchers have developed specialized scoring schemes based on the careful examination of the types of errors made by different subject groups. For example, Rey (1941) and Osterrieth (1944) were interested in the coarse dichotomy between spatial details and structural components, and recommended attention to organizational qualities of reproduction (such as the drawing order) rather than specific item errors. The Boston Qualitative Scoring System (BQSS; Stern et al., 1994) made the component hierarchies more explicit and added qualitative measures such as “fragmentation” and “planning.” On the other hand, Loring et al. (1988) developed novel classifications of specific types of spatial distortions (such as mislocations of particular elements of the ROCF, or additions of certain lines) that could discriminate between left and right epilepsy patients. Waber and Holmes (1985) studied the drawings of children using sophisticated evaluation metrics and statistical methods, determining sets of spatial features that created meaningful developmental classifications in concordance with subjective evaluations. In this spirit, we also propose new error types based on observations culled from the peripheral drawings.

## Methods

### Observers

Eight observers participated in the experiment. Observers were art students recruited from an art school and were naive to the purpose of the experiment. Art students were used since they would presumably be more able to accurately render their percepts due to drawing proficiency. All observers reported normal or corrected-to-normal visual acuity. The experiments were carried out according to ethical standards specified in the Declaration of Helsinki and were approved by the Ethics Committee of KU Leuven. Participants gave informed consent prior to beginning the experiment.

### Apparatus

**Figure 1.**
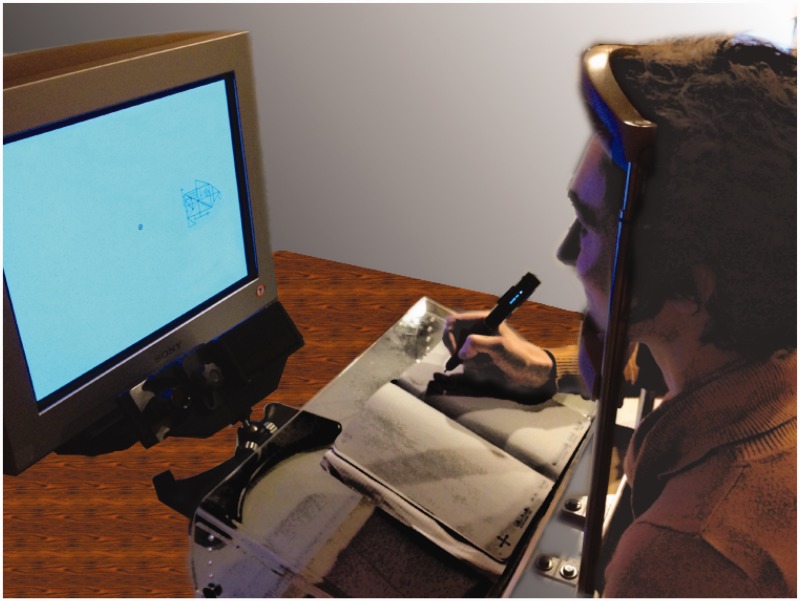
Experimental setup, showing (from left to right) the CRT monitor, eye tracking camera, drawing board, booklet, drawing pen, and head/chin rest.

Stimuli were presented on a Sony Trinitron GDM-F520 CRT monitor with a resolution of 1152 by 864 pixels and a refresh rate of 120 Hz. Subjects were seated 57 cm from the monitor, with their heads supported by a chinrest and headrest. Eye movements were monitored using an EyeLink 1000 eye tracker (SR Research Ltd., Mississauga, Ontario) at a 1000 Hz sampling rate. In front of the chinrest, there was an elevated drawing board, with the drawing booklet set on top. Subjects were able to move their gaze between the screen and the drawing booklet without head movements, and drew in the booklet using an electronic pen. The use of an electronic pen allowed immediate visual feedback of drawings, unlike traditional drawing tablets. The experimental setup is shown in Figure 1. The experiment was programmed in MATLAB (Mathworks, Natick Massachusetts, USA) using the Psychophysics toolbox (Brainard, 1997).

### Stimulus

**Figure 2.**
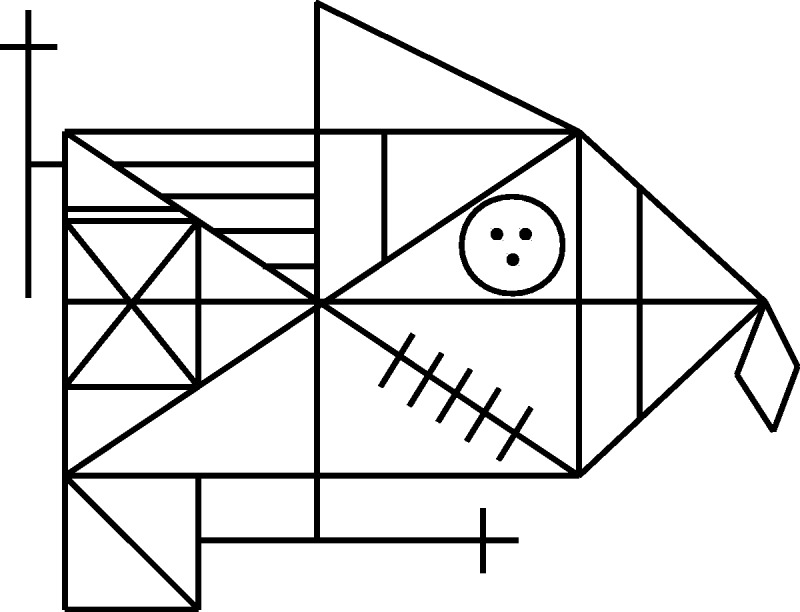
The Rey-Osterrieth Complex Figure.

The stimulus presented to the subjects was an 823 × 615 pixel bitmap of the ROCF subtending 9° by 7° (see Figure 2). Subjects were allowed to move their gaze between the screen and drawing booklet as often as they wanted. The stimulus was only presented when observers fixated the central fixation dot. First, subjects were shown the stimulus centered at 12° in the right visual field and were instructed to draw as accurately as possible how the stimulus appeared, that is, how it looked. After finishing the first drawing, subjects were shown the stimulus centered at 6° in the right visual field and created a new drawing based on this presentation. Finally, subjects were allowed to freely view the figure with their foveal vision, constituting a normal copy task. Subjects S2 and S7 did not perform the foveal condition and are omitted from the respective analyses. The order of eccentricities was used for all subjects in order to keep them as naive as possible about the presented figure.

The drawings were scored using two different scoring metrics by two independent experienced raters, naive to the purpose of the experiment. The scorers evaluated each drawing based on the original Osterrieth scoring system (Osterrieth, 1944) and the BQSS (Stern et al., 1994).

### Scoring Measures

#### Spatial elements: Osterrieth scoring

**Figure 3.**
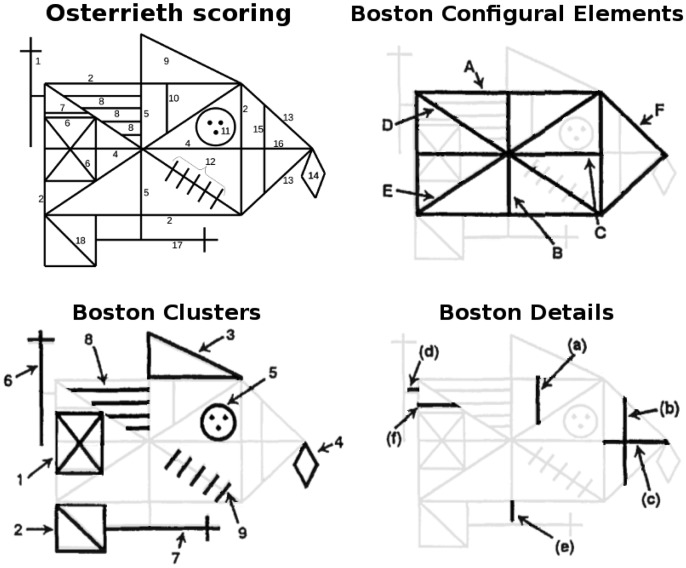
Osterrieth and Boston scoring systems. For the Osterrieth system (upper left), each numbered spatial element is given a score as described in the text. The Boston system (remaining three panels) is composed of three types of feature categories. For each of the 3 categories, scores from constituent elements are aggregated and counted based on accuracy and placement.

The original Osterrieth scoring system divides the figure into 18 spatial elements, as shown in Figure 3. Each element is given a score of 0, 1/2, 1, or 2. A score of 2 indicates correct reproduction and placement. A score of 1 indicates either of two possibilities: correct reproduction, but improper placement; or a distorted reproduction placed correctly. A score of 1/2 indicates items that are recognizable but possess both distorted appearance and improper placement. Zeros indicate items that are completely absent.

Specific guidelines exist for scoring each item (Osterrieth, 1944; Taylor, 1969; summarized by Duley et al., 1993). For example, for the circle with the three dots, there are prescribed penalties for “resembling a face,” and “incorrect placement of dots.” For the five parallel slanted lines, there are penalties for not crossing the large diagonal, being too long or too short, and so forth.

#### Boston Qualitative Scoring System

Each drawing was also evaluated using the BQSS. In this system, the drawing is divided into three categories of spatial elements: configural elements, clusters, and details (see Figure 3). Each of these categories is scored in aggregate, including evaluations of presence of the constituent elements, accuracy, and correct placement, all on a 1 to 5 scale. Global characteristics of the drawings, such as “neatness” and “fragmentation” are also scored.

### Statistical Analyses

The primary methods we used to perform post hoc statistical testing comprised parametric and nonparametric Monte Carlo methods. Specifically, observed data were resampled (with replacement), generating new data for use in computing *p*-values and confidence intervals. These analyses were performed in IPython with the Numpy/Scipy libraries (Oliphant, 2007; Pérez & Granger, 2007).

## Results

**Figure 4.**
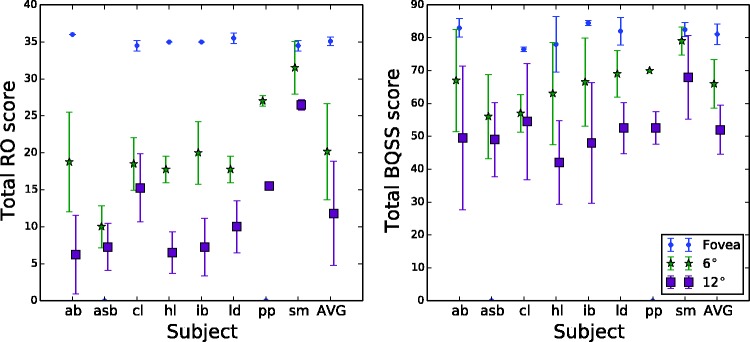
Total scores for each observer at all three eccentricities. Points and error bars indicate mean and standard deviation between raters. Right-most columns shows mean and standard deviation across subjects.

A summary of the total scores for each of the two major scoring systems is given in Tables 1 and 2, respectively. Individual participant data are plotted in the two panels of Figure 4. As expected, subjects performed nearly perfectly (35.1/36 for RO and 81.1/82 for BQSS) when they were allowed to use their fovea to view the figure. Total scores declined in the periphery. The correlation coefficient between the two raters for the summed scores was 0.97 for the Osterrieth system and 0.94 for the Boston system.

**Table 1.** Summary of Total Osterrieth Scores at Each Eccentricity.

Eccentricity	Score (mean)	Standard deviation	Range
Fovea	35.1	0.534	34.5–36
6°	20.2	6.07	10–31.5
12°	11.8	6.57	6.25–26.5

**Table 2.** Summary of Total BQSS Scores at Each Eccentricity.

Eccentricity	Score (mean)	Standard deviation	Range
Fovea	81.1	2.85	76.5–84.5
6°	65.9	6.94	56–79
12°	52	7.02	42–68

BQSS: Boston Qualitative Scoring System.

**Figure 5.**
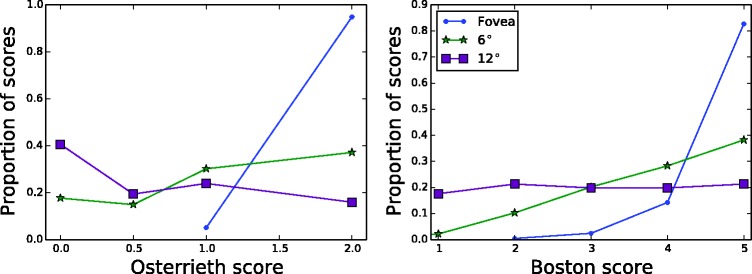
Distribution of scores at each eccentricity for each of the two rating systems. Dots represent the proportion of all scores for an eccentricity that possess the given rating, across both raters and all feature types.

The distributions of the individual element scores for each of the three eccentricities are shown in Figure 5. Most elements at the fovea are scored perfectly (2 for Osterrieth and 5 for BQSS), with a shift in the entire distribution to lower scores as the figure is placed further in the periphery. The non-Gaussian shapes of these distributions are an additional motivation for the use of Monte Carlo methods for determining confidence intervals.

**Figure 6.**
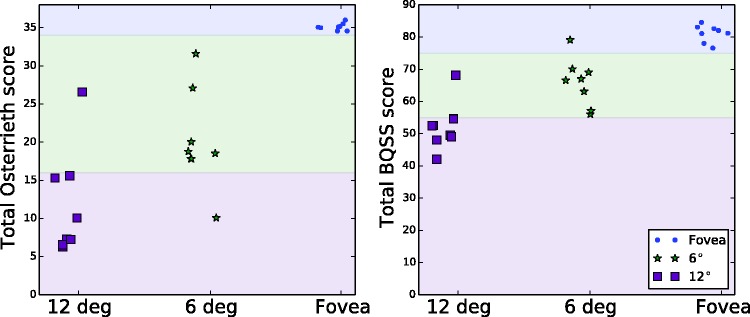
Data of Figure 4, re-ordered by eccentricity to better show classification boundaries. Each dot represents the total score (averaged across raters) for a subject at a given eccentricity.

As shown by Figure 4, the data separate fairly well based on their total scores. For each scoring system, a simple classifier can be constructed that thresholds the total score and estimates the eccentricity. For the Osterrieth scoring system, scores below 16 are estimated to result from 12°, scores above 34 are estimated to be from the fovea, and anything in between is assumed to be from 6°. This classifier is 95% accurate. It misclassifies S8’s 12° score as a 6° score and S2’s 6° score as a 12° score. A classifier based on the BQSS scores is similarly effective, with thresholds of <55 (for 12°) and >75 (fovea). Only the scores of S8 are outside the range—these scores are significantly higher than those of the other subjects. Figure 6 illustrates the classification boundaries by replotting the data from Figure 4.

**Figure 7.**
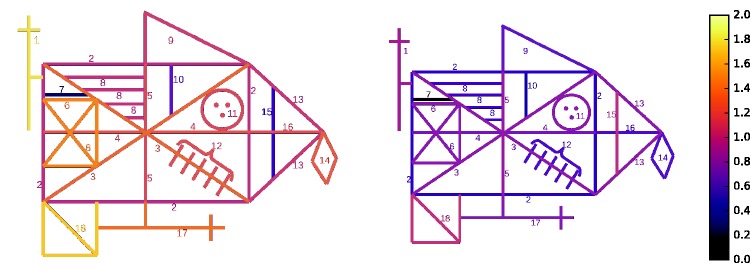
Graphical depiction of average feature scores listed in Table 3. Left panel shows 6° results, and right panel shows 12° results. Brighter colors indicate higher scores.

Quantifiable differences in the drawings reflect the influence of presentation eccentricity on drawing. Next, spatial features from the Osterrieth scoring scheme are analyzed. Table 3 lists the average score for each feature across observers at all three retinal locations, averaged across the two raters. Figure 7 illustrates these data by showing the average score across observers and raters for each spatial feature. Table 4 lists the scores from the Boston system at each of the three eccentricities.

**Table 3.** Osterrieth Feature Scores Averaged Across Raters.

Feature	Fovea	6°	12°
O1	1.92 ± 0.29	1.81 ± 0.40	0.91 ± 0.64
O2	2.00 ± 0.00	1.00 ± 0.80	0.38 ± 0.62
O3	2.00 ± 0.00	1.31 ± 0.57	0.81 ± 0.66
O4	2.00 ± 0.00	1.22 ± 0.80	0.69 ± 0.85
O5	2.00 ± 0.00	1.16 ± 0.65	0.84 ± 0.87
O6	2.00 ± 0.00	1.44 ± 0.63	0.78 ± 0.80
O7	1.67 ± 0.49	0.22 ± 0.52	0.12 ± 0.34
O8	2.00 ± 0.00	1.03 ± 0.62	0.44 ± 0.40
O9	2.00 ± 0.00	1.09 ± 0.58	0.59 ± 0.66
O10	2.00 ± 0.00	0.56 ± 0.87	0.31 ± 0.70
O11	2.00 ± 0.00	1.16 ± 0.70	0.81 ± 0.57
O12	1.92 ± 0.29	1.19 ± 0.51	0.50 ± 0.61
O13	1.58 ± 0.51	1.06 ± 0.63	0.81 ± 0.63
O14	2.00 ± 0.00	1.25 ± 0.77	0.53 ± 0.62
O15	2.00 ± 0.00	0.41 ± 0.71	1.00 ± 0.88
O16	2.00 ± 0.00	1.19 ± 0.91	0.50 ± 0.82
O17	2.00 ± 0.00	1.34 ± 0.62	0.75 ± 0.63
O18	2.00 ± 0.00	1.72 ± 0.52	1.03 ± 0.74
Mean	1.95 ± 0.12	1.12 ± 0.40	0.66 ± 0.25

*Note.* Numbers indicate average and standard deviation across observers for each eccentricity. See Figure 3 for feature key.

**Table 4.** BQSS Scores Averaged Across Raters.

Feature	Fovea	6°	12°
B1 (Configural elements presence)	5.00 ± 0.00	4.31 ± 0.87	2.94 ± 1.06
B2 (Configural elements accuracy)	4.83 ± 0.39	3.31 ± 1.14	2.31 ± 1.20
B3 (Cluster presence)	5.00 ± 0.00	4.50 ± 0.63	2.88 ± 1.15
B4 (Cluster accuracy)	4.67 ± 0.49	3.12 ± 0.89	1.88 ± 0.96
B5 (Cluster placement)	4.92 ± 0.29	3.94 ± 0.93	2.94 ± 1.34
B6 (Detail presence)	4.92 ± 0.29	2.75 ± 0.93	1.81 ± 0.83
B7 (Detail accuracy)	4.58 ± 0.51	3.50 ± 1.59	2.25 ± 1.69
B8 (Fragmentation)	5.00 ± 0.00	4.19 ± 0.83	2.75 ± 1.24
B9 (Planning)	4.92 ± 0.29	3.44 ± 1.09	1.81 ± 1.17
B10 (Size reduction)	4.83 ± 0.39	3.94 ± 1.18	3.25 ± 1.53
B11 (Vertical expansion)	4.75 ± 0.45	4.44 ± 0.89	4.06 ± 1.06
B12 (Horizontal expansion)	4.50 ± 0.67	4.38 ± 0.81	4.19 ± 0.98
B13 (Rotation)	4.83 ± 0.39	4.38 ± 1.26	4.81 ± 0.40
B14 (Perseveration)	4.92 ± 0.29	4.12 ± 1.26	4.06 ± 0.85
B15 (Confabulation)	4.92 ± 0.29	4.31 ± 0.70	3.62 ± 0.89
B16 (Neatness)	4.83 ± 0.39	3.75 ± 1.00	2.94 ± 1.29
B17 (Asymmetry)	4.00 ± 1.18	3.56 ± 1.41	3.50 ± 1.10
Mean	4.79 ± 0.25	3.88 ± 0.52	3.06 ± 0.89

BQSS: Boston Qualitative Scoring System. Numbers indicate average and standard deviation across observers for each eccentricity.

To determine which scores differed significantly from other scores at each of the two peripheral eccentricities, we performed Monte Carlo simulations of the data. Simulated feature scores were drawn with replacement from the observed per-rater scores at each eccentricity. Most of the feature occurrence rates did not differ from the mean. However, some features did have a frequency that was outside the 95% confidence intervals determined by the Monte Carlo procedure. Table 5 summarizes the results of this analysis, showing only those features that were present in the drawings with a statistically different frequency (i.e., higher or lower than the 95% confidence intervals for that eccentricity).

**Table 5.** For Each Scoring System and Eccentricity, the Features That Are Listed Are Those With a Proportion That Differs Statistically From the Population Mean.

Feature type	Eccentricity	Smaller than CI	Larger than CI
Osterrieth	6°	#7, #10, #15	#1, #18
	12°	#7	
BQSS	6°	#6 (detail presence)	
	12°	#6, #4 (cluster accuracy)	#11, #12, #13, #14

*Note.* BQSS: Boston Qualitative Scoring System.These features were either higher or lower than the 95% confidence intervals determined by Monte Carlo simulation using all feature scores from all subjects and both raters. The first two rows indicate Osterrieth features (see Figure 3), and the last two rows indicate BQSS features (see text).

For the Osterrieth features (see Figure 3), identification of the small line directly above the left-side square (Feature #7) differed from the other features, at both 6° and 12°. At 6°, the small vertical line in the top-right corner (#10) and the larger interior vertical line on the right (#15) are also significantly omitted. At 6°, the left-side cross (#1) and the left-bottom square (#18) are identified more correctly than other features.

For the Boston scores, “detail presence” is the only score which differs significantly at 6°. At 12°, this score, as well as “cluster accuracy” is significantly outside the 95% confidence intervals. Several features are significantly greater than the other scores at 12°. Those are vertical and horizontal expansion, rotation, and perseveration (inappropriate repetitions). These error types did not occur as often as the other errors.

Previous qualitative scoring systems were based on careful characterization of error types from different subject populations. For example, Loring et al. (1988) identified particular types of spatial errors (e.g., “misplacement of upper left cross”) that were diagnostic of the hemispheric lateralization of seizures in epileptic patients. Motivated by this approach, we identified new types of errors observed in the peripheral drawings. Table 6 lists the error types and indicates which subjects exhibited the errors at each eccentricity. None of these errors occurred in foveal drawings. The errors fall into two broad categories: depiction of an inaccurate number of adjacent similar items (number errors) and specific spatial deformations (distortion errors).

**Table 6.** Feature Errors for a New Qualitative System Based on the Observed Error Patterns.

Error type	Feature	6°	12°
Number	Incorrect number of dots in circle	S1–S5	S1–S8
	Diagonal lines under-counted	S1, S4, S5, S6, S7	S2, S4, S7
	Horizontal lines under-counted	S1, S2, S4, S6, S7	S1–S8
	Extra down/rightward diagonal element	S3, S7	S1, S2, S3
Distortion	Diagonal lines become zig-zag “staircase”		S1, S5, S6, S7
	Rounded upper right-hand perimeter	S2	S1, S2, S5–S7

*Note.* Subjects exhibiting the error at each eccentricity are indicated.

**Figure 8.**
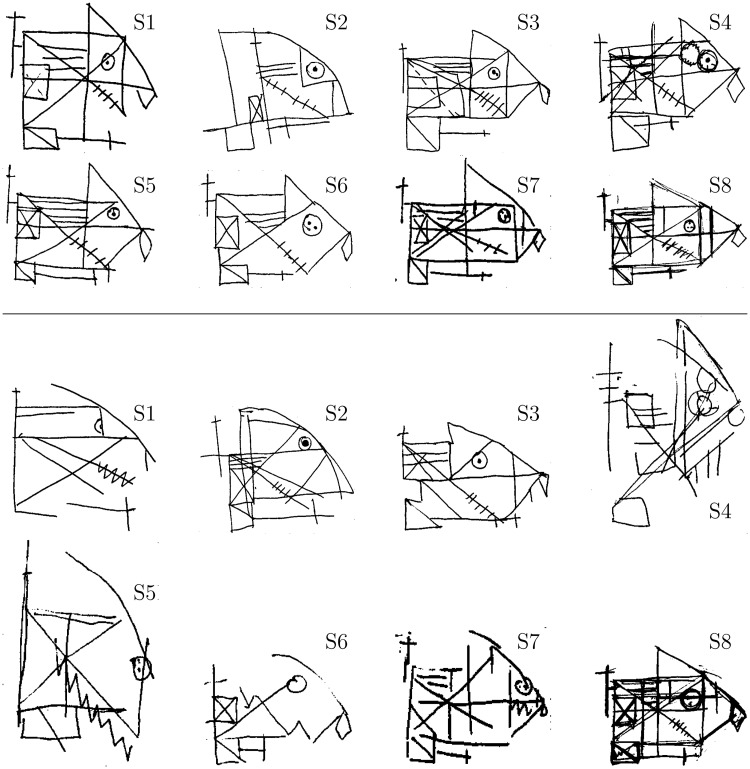
The top two rows reproduce the drawings made when subjects viewed the figure at 6°, and bottom two rows are from 12° presentations. Drawings were spatially scaled for presentation consistency.

Figure 8 reproduces all subjects’ drawings from the two peripheral locations. Note that drawings are not shown in equal scale. For display purposes, each drawing was digitally scaled to yield similar sizes.

## Discussion

As far back as Ptolemy and Alhazen, it has been noted that peripheral vision lacks the clarity of foveal vision (Strasburger & Wade, 2015). Since then, researchers have sought to more fully characterize the nature of the deficiencies in peripheral perception. Experiments with traditional psychophysical methods have yielded important findings about the effects of spatial undersampling, the loss of phase information, or the deleterious effect of neighboring contours on identification of a target (for a review of pattern vision in the periphery, see Strasburger et al., 2011).

Increasingly, however, researchers are interested in determining more fully the phenomenological nature of peripheral distortions. What do objects actually “look like” in the periphery? For example, the common intuition is that peripheral objects should appear blurrier than foveal objects, due to the declining sampling resolution in the periphery. Paradoxically, the opposite is true: In matching experiments, subjects perceive peripheral objects to be sharper than corresponding foveal objects (Galvin, O’Shea, Squire, & Govan, 1997). Hence, alternate characterizations of peripheral appearance, such as localized spatial disorder (Koenderink & van Doorn, 2000), are needed. Furthermore, composite nonfoveal objects like words are ambiguous in very specific ways related to mislocalization of features and symbols, as described by Korte (1923) and further clarified by Strasburger (2014). Even before Korte’s (1923) treatise, scientists such as James Jurin and Alhazen were aware of this *indistinct* mode of vision that hinders recognition of compound objects. However, the categorical difference in phenomenology between indistinct vision and the straightforward loss of resolution (as apparent even with simple objects) has not always been appreciated (Strasburger & Wade, 2015).

An understanding of the phenomenology of peripheral vision is crucial, particularly for building models designed to capture the so-called *metamers* of spatial vision (Koenderink & van Doorn 1996). Impoverished stimuli and limitations of forced-choice methods may mask the shortcomings of models of peripheral vision. For example, a recent study demonstrated that highly sensitive psychophysical methods are necessary to evaluate the peripheral information available to observers (Wallis, Bethge, & Wichmann, 2016). Specfically, these authors found that subjects could make better discriminations than those predicted by texture-based models of crowding (Balas et al., 2009; Freeman & Simoncelli, 2011). Here, we chose to use the method of drawing to directly capture the visual distortions in peripheral percepts. For task standardization, we used a mature method from neuropsychology that has been employed for over 50 years.

Using the Rey–Osterrieth drawing task and standard scoring methods, we showed how accurate reproduction of spatial features predictably declined as the target was presented further in the visual periphery. Furthermore, the more foveal elements of each drawing, such as the left-most cross (Feature #1), were often more accurately depicted in the drawings. However, eccentricity was not the only factor involved. Specifically, in agreement with crowding explanations, the proximity and similarity of an element to nearby features also had an impact on difficulty. For example, the detection rate of Feature #7 was relatively low, presumably because it is very close to the upper line of Item #6, which is similar in length and of the same orientation as Feature #7. Korte (1923) noted that features which were not integrated into an overall form were more likely to elicit ambiguous percepts. This would be the case for Feaures #7, #10, and #15 of the ROCF, which were often excluded by our subjects. On the other hand, features on the exterior of the figure, such as the cross mentioned earlier (Feature #1), or Feature #14, which hangs off of the right side of the figure, are flanked with fewer elements than interior features, and are identified more accurately. To precisely decouple the role of eccentricity, crowding and grouping in the ROCF would require additional experiments, such as showing a mirror-image of the figure.

The outline of the shape was often identified correctly, although interestingly the upper-right corner often assumed a curved shape. Most drawings retained overall configural structure (the outline rectangle and its diagonal elements) at 6°. At 12°, only some of the drawings retained the configural structure. Interestingly, Feature #15 (the medium-sized vertical line in the right-hand of the figure) was identified poorly at 6° but more accurately at 12°. Looking at the drawings, there is an intuitive explanation. At 6°, the outline rectangle was identified relatively accurately, but this vertical line was often missed. At 12°, the outline rectangle was typically distorted or absent, but there was some vertical line feature for most subjects. This vertical line was generally scored as Feature #15.

Observations from several other components of the figure strengthen the proposal that it is difficult to individuate items in structured repeating patterns (Intriligator & Cavanagh, 2001). For example, the sets of parallel lines were recognizable in the drawings, but typically undercounted. This is in contrast to most previous observations with the figure in neuropsychological settings, which have observed additional parallel lines, especially with damage to the right-temporal lobe (Loring et al., 1988). This reduction in featural elements agrees well with our recent account of crowding (Sayim & Wagemans, 2013; see also Liu & Arditi, 2000). Another example is the circle and its constituent dots. The overall pattern was often depicted, but the particular number of dots and their spatial relationships were often lost.

Clearly, there was great individual variability in the drawings, from being quite sparse and unusual at 12° (Subjects S1, S4, S5, and S6) to being nearly perfect at both eccentric locations (Subject S8). It has previously been noted that subjects may have very different crowding zone extents (Pelli et al., 2007) and can exhibit other idiosyncratic individual differences in the strength of crowding effects, such as the “inner/outer” anisotropy due to the flanker position relative to the target and the fovea (Petrov & Meleshkevich, 2011). Furthermore, when the visual input is highly ambiguous, which is often the case for peripheral objects, factors such as imagination may affect visual perception more than for direct vision. Some subjects reported that the figure looked like a “face” or a “fish,” which could have influenced perception by “filling-in” missing details. The extent to which low-level perception may be affected by cognition is an active and controversial topic (Firestone & Scholl, 2015; Pylyshyn, 1999). It has also been proposed that the processes involved in depiction utilize schemata influenced by culture, training, and experience (Gombrich, 2000). At minimum, previous exposure to this particular figure may have enhanced the reproduction for some subjects.

Since drawing is a learned proficiency, we opted to use student artists for this study. This population should be more able to accurately depict their percepts, counteracting the sources of noise inherent in the procedure such as motor ability or visual memory limitations. On the other hand, the visual perception of artists may differ from that of average observers. For example, it has been observed that skilled artists may differ in aspects of visual cognition (Chamberlain & Wagemans, 2015, 2016; Kozbelt, 2001; Perdreau & Cavanagh, 2013a, 2014, but see Perdreau & Cavanagh, 2013b). Chamberlain, McManus, Brunswick, Rankin, and Riley (2015) found that in a large population of student artists greater drawing ability was correlated with higher ROCF copy scores, and Gallagher and Burke (2007) found differences in raw ROCF scores based on factors such as IQ and gender. Future studies with a more general population would be required to investigate the generality of our observations, albeit with the cost of reduced overall drawing skills.

Interestingly, our foveal results (averaging 35.1 out of 36 on the Osterrieth rating) seem better than those found in a recent large study that also used artists as observers (Chamberlain et al., 2015), which had an average Osterrieth rating of 31.88. There are several possible reasons for the difference. While it could be that our sample of artists was biased toward more skilled artists, we suggest that the difference in time allotment underlies this finding. In Chamberlain et al. (2015), observers were only given 4 min to copy the figure, whereas we allowed unlimited time.

A further methodological aspect of our experiment differs from typical crowding studies. In this experiment, viewing time was unlimited and subjects were permitted to view the stimuli as many times as necessary by allowing unrestricted gaze shifts between the drawing book and the monitor. In most crowding experiments, display duration is brief (usually 100–200 ms), ostensibly to avoid undesired eye movements to the peripheral target. Stimulus duration has a measurable effect on the magnitude and extent of crowding (Chung & Mansfield, 2009; Tripathy & Cavanagh, 2002; Tripathy, Cavanagh, & Bedell, 2014), though there are few studies testing unlimited duration with gaze-contingent displays (i.e., Wallace, Chiu, Nandy, & Tjan, 2013). Importantly, the stability and persistence of crowded percepts has not been previously characterized, a topic that warrants future direct study.

Finally, besides a better theoretical understanding of how peripheral visual perception differs from foveal perception, there is a translational aspect to this study as well. Age-related macular degeneration is a condition whereby the fovea becomes unusable due to disease, is a leading cause of visual impairment (Congdon, 2004; de Jong, 2006), and is growing in prevalence amongst older adults (Congdon, 2004). Since peripheral viewing is mandatory for subjects with a disorder such as this, visual crowding is a limiting factor (Chung, 2014). Proper application of the ROCF test in such a patient group requires an understanding of how perception of the ROCF changes in the periphery. For example, the ROCF has been shown to be sensitive to the laterality of stroke (Binder, 1982; Lange, Waked, Kirshblum, & DeLuca, 2000). For a stroke patient with age-related macular degeneration, ROCF results may differ from the normative population due to visual factors rather than neurocognitive factors.

While the difficulties processing complicated forms in the periphery have been studied by researchers for nearly a century, it is only recently that the specific characteristics of the form and shape degradations are becoming understood. We believe that more diverse stimuli are crucial to pursue this goal, and the use of the ROCF opens up promising new avenues to explore this question. The task of drawing presents a way for subjects to give a precise account of their peripheral percepts, providing a rich amount of information complementing forced-choice methods.
